# Suppression of *Propionibacterium acnes*-Induced Skin Inflammation by *Laurus nobilis* Extract and Its Major Constituent Eucalyptol

**DOI:** 10.3390/ijms20143510

**Published:** 2019-07-17

**Authors:** Eun Hye Lee, Jin Hak Shin, Seon Sook Kim, Ji-Hye Joo, Eunmi Choi, Su Ryeon Seo

**Affiliations:** 1 Department of Molecular Bioscience, College of Biomedical Science, Institute of Bioscience & Biotechnology, Kangwon National University, Chuncheon 24341, Korea; 2 R&D Center, Greensolutions Co., Chuncheon 24342, Korea

**Keywords:** *Laurus nobilis* L., inflammation, eucalyptol, *Propionibacterium acnes*, proinflammatory cytokines, NF-κB

## Abstract

Acne is an inflammatory skin disorder in puberty with symptoms including papules, folliculitis, and nodules. *Propionibacterium acnes* (*P. acnes*) is the main anaerobic bacteria that cause acne. It is known to proliferate within sebum-blocked skin hair follicles. *P. acnes* activates monocytic cell immune responses to induce the expression of proinflammatory cytokines. Although the anti-inflammatory function of the *Laurus nobilis* (*L. nobilis*) extract (LNE) on several immunological disorders have been reported, the effect of LNE in *P. acnes*-mediated skin inflammation has not yet been explored. In the present study, we examined the ability of the LNE to modulate the *P. acnes*-induced inflammatory signaling pathway, and evaluated its mechanism. LNE significantly suppressed the expression of *P. acnes*-mediated proinflammatory cytokines, such as IL-1β, IL-6, and NLRP3. We also found that LNE inhibited the inflammatory transcription factor NF-κB in response to *P. acnes*. In addition, eucalyptol, which is the main constituent of LNE, consistently inhibited *P. acnes*-induced inflammatory signaling pathways. Moreover, LNE significantly ameliorated *P. acnes*-induced inflammation in a mouse model of acne. We suggest for the first time that LNE hold therapeutic value for the improvement of *P. acnes*-induced skin inflammation.

## 1. Introduction

Acne is a common skin inflammatory disorder in sebaceous glands that causes inflammation in the face, neck, back, chest, and shoulders. Acne occurs mainly in adolescence due to hormone imbalance, bacterial infections, food, and psychological stress [1]. *Propionibacterium acnes*, a commensal Gram-positive anaerobia, is thought to be the primary bacteria in acne development [2]. *P. acnes* overgrows in hair follicles and acts as an immunostimulator to secrete various proinflammatory cytokines that are crucial for the development of cutaneous inflammation [3]. The peptidoglycan cell wall of *P. acnes* has been proposed to activate monocytes to produce cytokines such as IL-1β, IL-8, and TNF-α, which cause granulomatous responses in inflammatory skin disorder [4,5]. *P. acnes* is recognized by the toll-like receptors TLR2 and TLR4 and triggers the expression of inflammatory mediators through activating MAP kinases (MAPKs) and nuclear factor-κB (NF-κB) [6,7].

Antibiotics, including tetracycline, erythromycin, and clindamycin, are commonly used for the treatment of *P. acnes*-induced inflammation [8]. Benzoyl peroxide (BPO), isotretinoin, and synthetic sulfone have recently been reported to be efficient in the treatment of acne [9,10,11]. However, current standard treatments have been shown to have adverse effects and poor efficacy. It is obvious that the development of therapeutic agents with low side-effects but high antimicrobial activity would be important for the treatment of acne.

*Laurus nobilis Linn.* has been cultivated in the Southern Mediterranean sea region and in Europe, as a vegetable and a traditional medicine [12]. *L. nobilis* has been reported to have antioxidant, antimicrobial, analgesic, anticonvulsant, and antifungal actions [13,14,15]. The dried leaves are widely used as a natural remedy to treat arthritis, rheumatism, asthma, and inflammation [16,17,18].

Although the inhibitory effect of *L. nobilis* extract (LNE) on diverse inflammatory responses have been reported, the role of LNE in *P. acnes*-mediated skin inflammation has not yet been reported. Therefore, we examined the potential effects of LNE in response to *P. acnes*, using in vitro and in vivo mouse experimental models. We found that LNE and its major constituent eucalyptol effectively exert anti-inflammatory functions by suppressing *P. acnes*-induced inflammatory signaling pathways.

## 2. Results

### 2.1. Dose Optimization of LNE in Bone Marrow-Derived Macrophages (BMMs).

**Figure 1:**
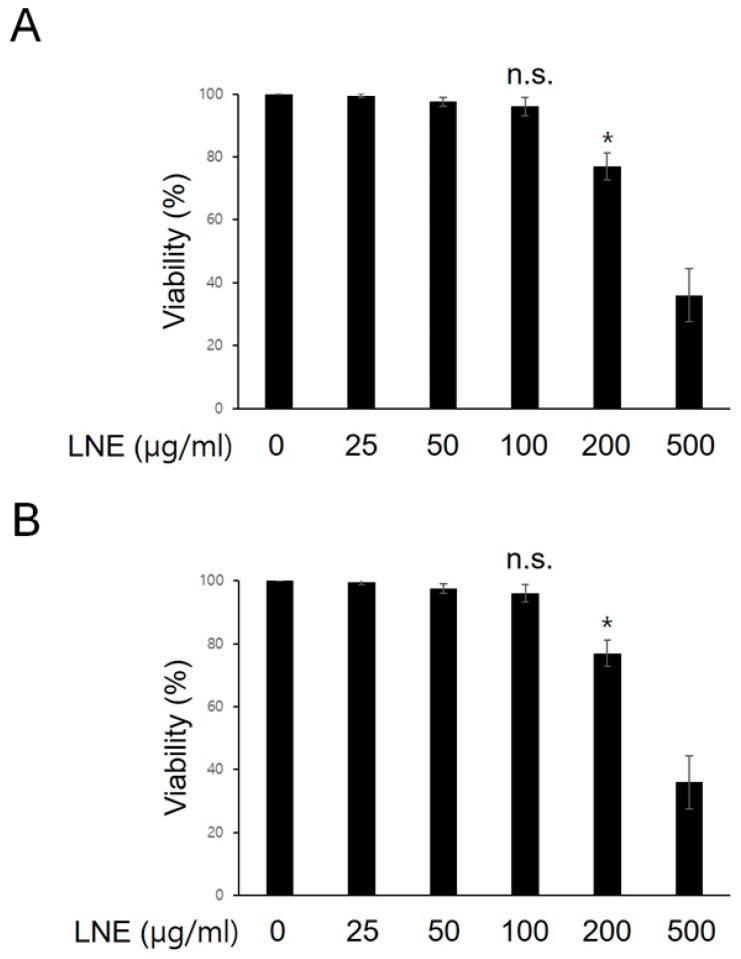
Determination of *Laurus nobilis* (*L. nobilis*) extract (LNE) concentrations in Bone Marrow-Derived Macrophages (BMMs). Cells were treated with different concentrations of LNE for 12 h, and the cell viability was measured using either the Trypan blue dye exclusion assay (**A**) or the MTT assay (**B**). * *p* < 0.05, n.s.—non-significant.

To investigate the possible effect of LNE on *P. acnes*-induced inflammatory signaling pathways, we first determined the noncytotoxic concentration of LNE to BMMs. For this experiment, BMMs were incubated with increasing concentrations of LNE for 12 h, and cell viability was assessed using Trypan blue dye exclusion (Figure 1A). We found that LNE did not affect the viability of BMMs up to 100 μg/mL (Figure 1A). The measurement of viability by MTT assay consistently showed that the viability of BMMs was not affected by LNE up to 100 μg/mL (Figure 1B). Based on these results, we selected 25 and 50 μg/mL LNE, which appeared to be safe concentrations to use, and performed all subsequent experiments with it.

### 2.2. Inhibition of P. acnes-Induced Proinflammatory Cytokine Expression by LNE

**Figure 2:**
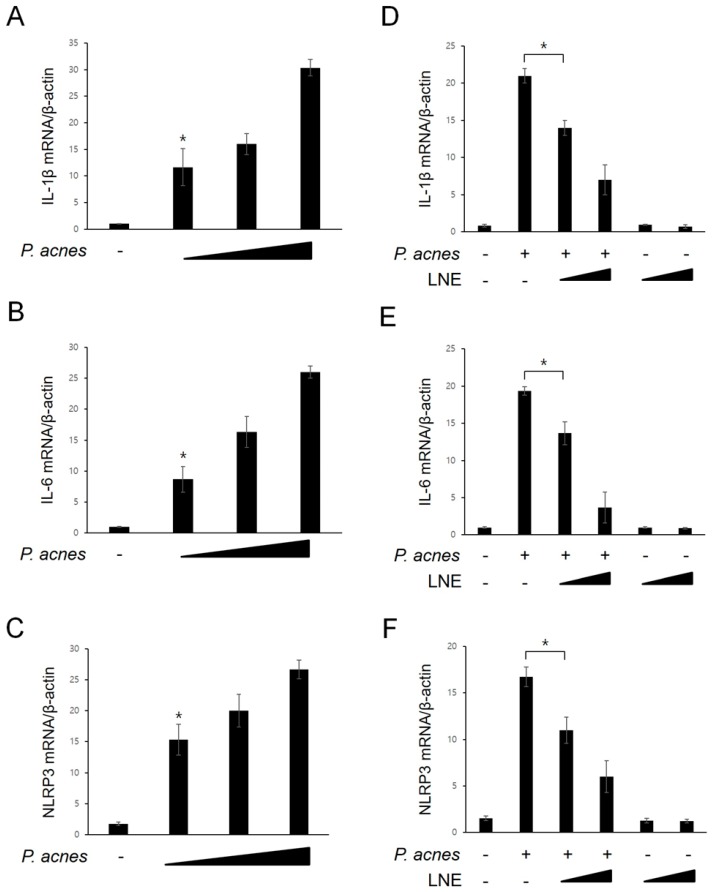
LNE inhibits *P. acnes*-induced proinflammatory cytokine expression. (**A**–**C**) BMMs were treated with increasing concentrations of *P. acnes* (1 × 10^5^, 1 × 10^6^ and 1 × 10^7^ CFU) for 6 h, and the mRNA (IL-1β, IL-6 and NLRP3) levels were analyzed using quantitative real-time PCR. (**D**–**F**) Cells were treated with LNE (25 and 50 μg/mL) for 30 min before *P. acnes* stimulation (1 × 10^7^ CFU) for 6 h. The mRNA (IL-1β, IL-6, and NLRP3) levels were analyzed using quantitative real-time PCR. * *p* < 0.05.

Next, we examined whether *P. acnes* was capable of inducing the expression of inflammatory mediators. For this experiment, BMMs were treated with increasing concentrations of *P. acnes*, and the mRNA levels of inflammatory mediators were monitored using quantitative real-time PCR (Figure 2A–C). As shown in Figure 2A,B, mRNA levels of proinflammatory cytokines, IL-1β and IL-6, were increased by *P. acnes* in a concentration-dependent manner. In addition, the NLRP3 inflammasome expression, which is induced by various cellular danger signals, was consistently increased in response to *P. acnes* (Figure 2C). We next determined the effect of LNE on the expression of these inflammatory mediators after exposure to *P. acnes* (Figure 2D–F). As shown in Figure 2D–F, IL-1β, IL-6, and NLRP3 mRNA expression levels in response to *P. acnes* treatment were significantly attenuated by LNE pretreatment in a concentration-dependent manner.

### 2.3. Inhibition of P. acnes-Induced NF-κB Activation by LNE

**Figure 3:**
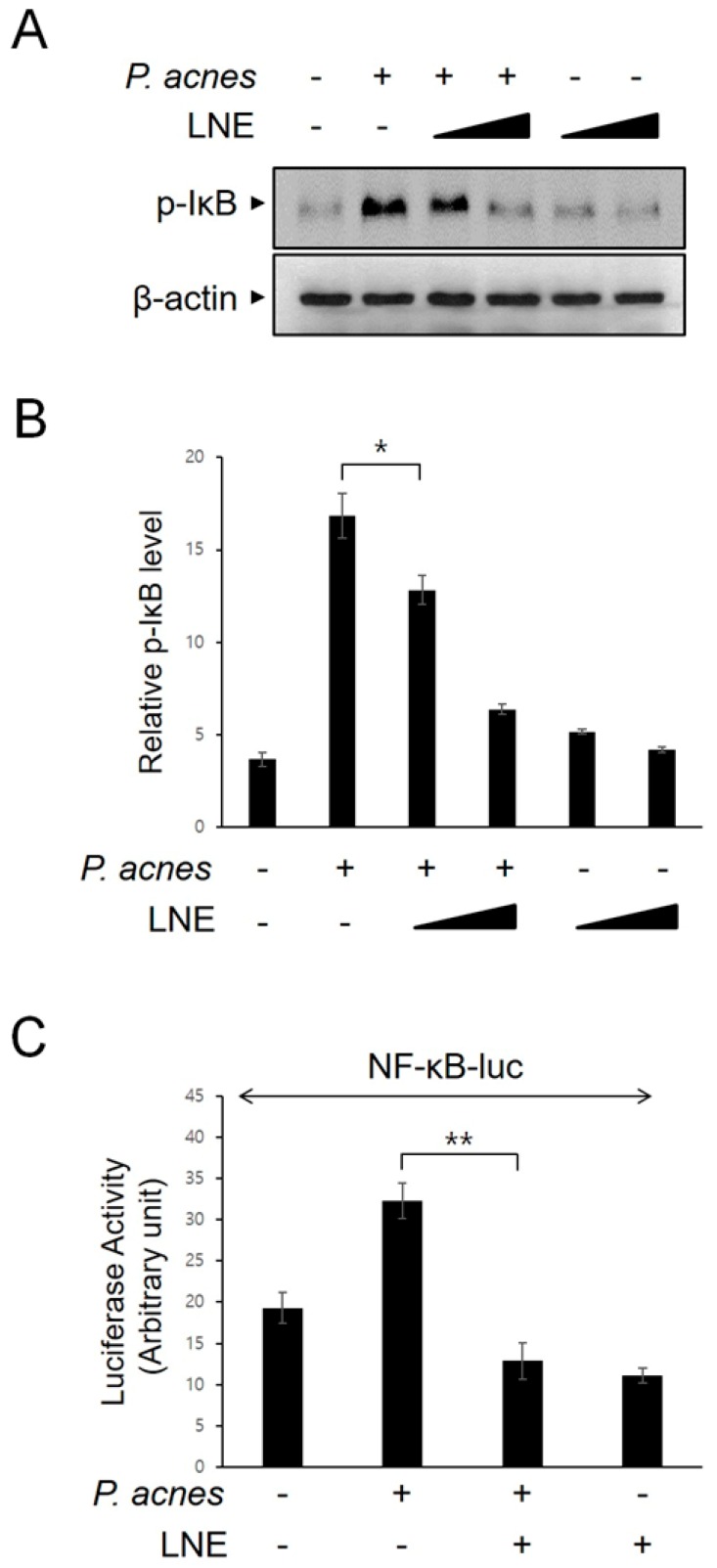
LNE inhibits the *P. acnes*-induced NF-κB signaling pathways. (**A**,**B**) BMMs were treated with LNE (25 and 50 μg/mL) for 30 min before *P. acnes* stimulation (1 × 10^7^ CFU) for 6 h. Phospho-IκB and GAPDH expression were detected by Western blot (**A**) and quantified (**B**). (**C**) The NF-κB-luciferase construct (NF-κB-Luc) was transfected into HEK293 cells and expressed for 24 h. The cells were treated with *P. acnes* (1 × 10^7^ CFU) in the presence or absence of LNE (50 μg/mL) for 12 h and then the luciferase activity was measured. * *p* < 0.05, ** *p* < 0.01.

We examined the effect of LNE on the activation of the nuclear factor-kappa B (NF-κB) transcription factor, which is known to be critical for the expression of various inflammatory mediators. We first monitored IκB phosphorylation, which unmasks NF-κB. LNE effectively suppressed IκB phosphorylation in BMMs, following *P. acnes* stimulation (Figure 3A,B). To further confirm the effect of LNE on *P. acnes*-mediated NF-κB signaling, NF-κB-dependent reporter analysis was performed (Figure 3C). Consistently, LNE significantly inhibited the NF-κB-dependent gene transcription in response to *P. acnes*.

### 2.4. Inhibition of P. acnes-Induced NLRP3 Inflammasome Activation by LNE

**Figure 4:**
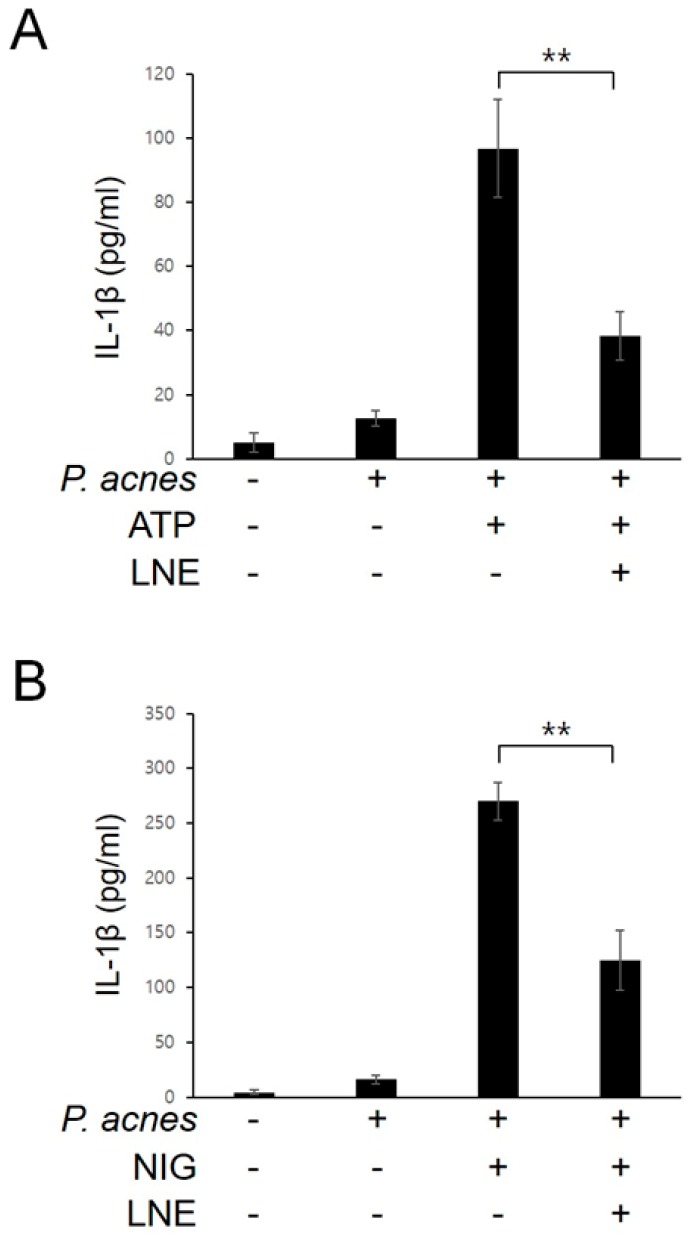
LNE inhibits the *P. acnes*-induced NLRP3 inflammasome activation. BMMs were primed with *P. acnes* (1 × 10^7^ CFU) for 3 h and then with LNE (50 μg/mL) treatment for 30 min before either ATP (5 mM) (**A**) or nigericin (NIG, 10 μM) (**B**) stimulation for 1 h. Secreted IL-1β was quantified using ELISA. ** *p* < 0.01.

Nod-like receptor pyrin domain-containing 3 (NLRP3) inflammasome regulates inflammation by triggering the maturation and secretion of IL-1β [19]. To investigate the effect of LNE on NLRP3 inflammasome activation, *P. acnes*-primed BMMs were treated with the well-known NLRP3 inflammasome activator ATP, which opens the P2X7 cation channel, in the presence or absence of LNE. As shown in Figure 4A, LNE suppressed ATP-driven IL-1β protein secretion. The reduction in active IL-1β secretion by LNE in response to ATP was further confirmed by another canonical NLRP3 inflammasome activator, nigericin, which functions directly as a K^+^/H^+^ exchanger on partitioning into intracellular organelles and plasma membranes (Figure 4B). Collectively, these results show that LNE inhibits *P. acnes*-primed NLRP3 inflammasome activation.

### 2.5. P. acnes-Induced MAPK Activation is not Inhibited by LNE

**Figure 5:**
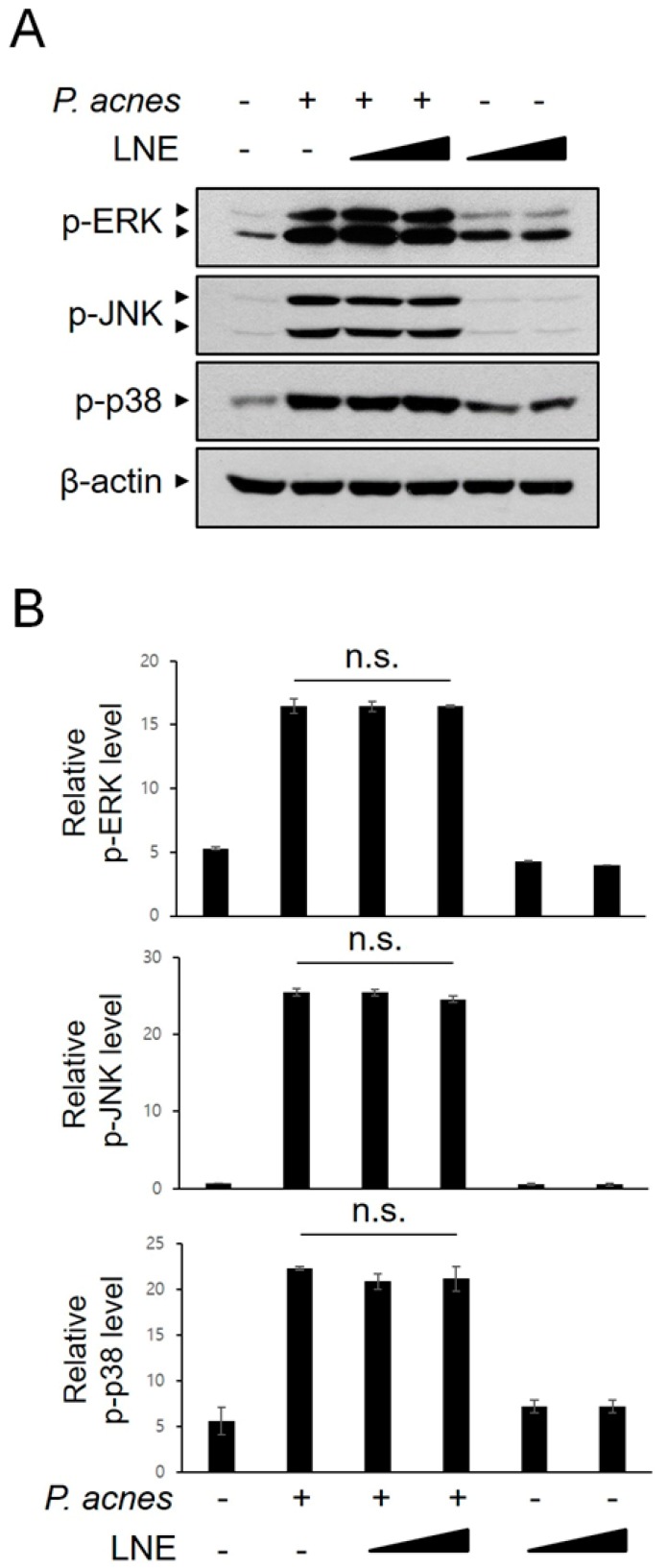
LNE has no effect on the *P. acnes*-induced MAPK signaling pathways. BMMs were treated with LNE (25 and 50 μg/mL) for 30 min before *P. acnes* stimulation (1 × 10^7^ CFU) for 6 h. Phosphorylated MAPKs and GAPDH expression were detected by Western blot (**A**) and quantified (**B**). n.s.—non-significant.

Activation of MAPKs, such as ERK, JNK, and p38 MAPK, is involved in the regulation of the downstream signaling cascade, including transcription factors and effectors, during the inflammatory response. We determined whether LNE modulates MAPK signaling in *P. acnes*-mediated inflammation. BMMs were treated with *P. acnes* in the presence or absence of LNE, and the activation of MAPKs was monitored using phospho-specific antibodies (Figure 5A,B). As shown in Figure 5A,B, LNE did not inhibit *P. acnes*-induced MAPK phosphorylation, suggesting that the activation of MAPKs is not involved in LNE-mediated anti-inflammatory effects.

### 2.6. Eucalyptol Inhibits P. acnes-Induced Inflammation

**Figure 6:**
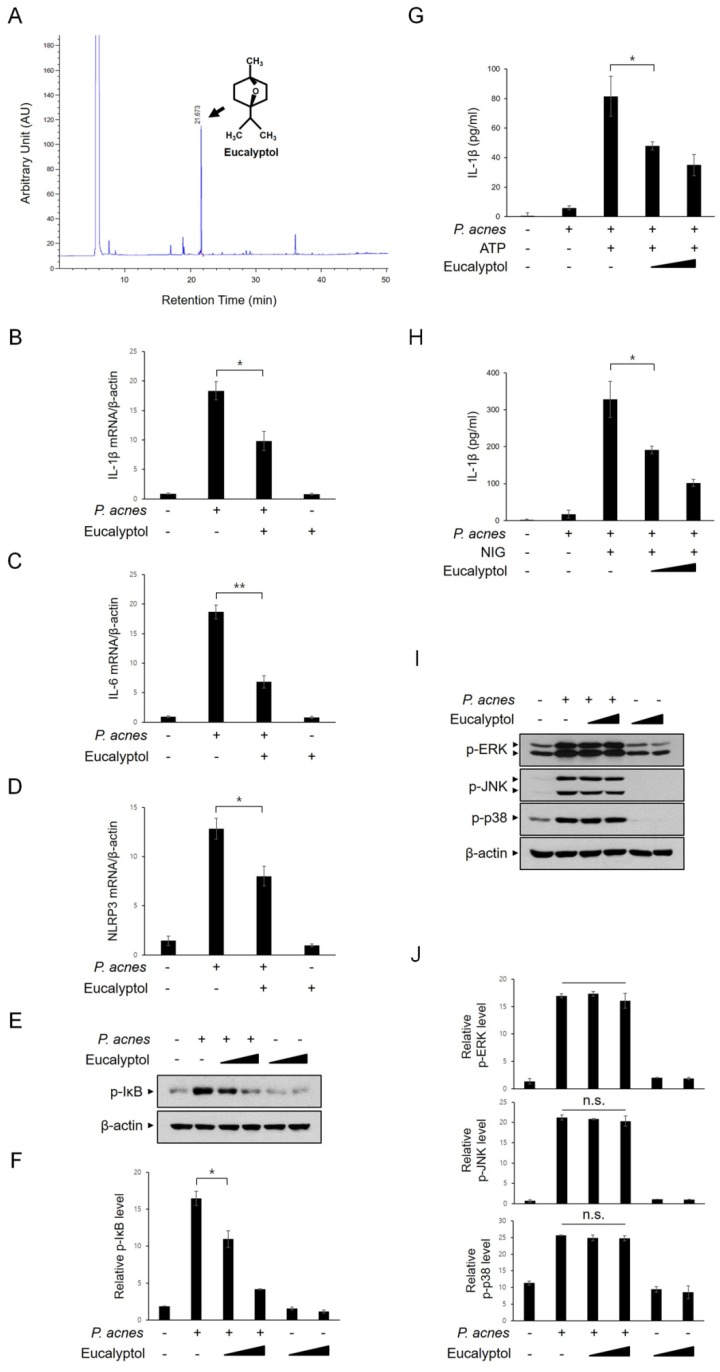
Eucalyptol inhibits *P. acnes*-induced inflammatory signaling pathways. (**A**) Isolation of eucalyptol. (**B**–**D**) BMMs were treated with eucalyptol (30 μM) for 30 min before *P. acnes* treatment (1 × 10^7^ CFU, 6 h). The mRNA (IL-1β, IL-6, and NLRP3) levels were determined using quantitative real-time PCR. (**E**,**F**) BMMs were treated with eucalyptol (10 μM and 30 μM) for 30 min before *P. acnes* stimulation (1 × 10^7^ CFU) for 6 h. Phospho-IκB and anti-GAPDH expression were detected by Western blot (**E**) and quantified (**F**). (**G**,**H**) BMMs were primed with *P. acne* (1.0 × 10^7^ CFU) for 3 h and then with eucalyptol (10 μM and 30 μM) for 30 min, followed by either ATP (5 mM) (**G**) or nigericin stimulation (NIG, 10 μM) (**H**) for 1 h. Secreted IL-1β was determined using ELISA (**I**,**J**). BMMs were treated with eucalyptol (10 μM and 30 μM) for 30 min before *P. acnes* stimulation (1 × 10^7^ CFU) for 6 h. Phospho-MAPKs and GAPDH expression were detected by Western blot (**I**) and quantified (**J**). * *p* < 0.05, ** *p* < 0.01, n.s.—non-significant.

We investigated which constituent of LNE is involved in the suppression of *P. acnes*-induced inflammatory pathways. Previously, several groups reported the isolation of eucalyptol as a main constituent of LNE [20,21]. Consistent with these reports, the presence of eucalyptol in our LNE was identified by GC–MS chromatogram (Figure 6A). Next, we monitored the effect of eucalyptol on the mRNA levels of inflammatory mediators in response to *P. acnes* using real-time PCR. *P. acnes*-induced increase in IL-1β, IL-6, and NLRP3 mRNA levels were significantly inhibited by the eucalyptol pretreatment (Figure 6B–D). IκB phosphorylation in response to *P. acnes* stimulation was consistently inhibited by eucalyptol in BMMs (Figure 6E,F). To determine the effect of eucalyptol on NLRP3 inflammasome activation, *P. acnes*-primed BMMs were treated with eucalyptol and ATP, and active IL-1β protein levels were measured using ELISA (Figure 6G). As shown in Figure 6G, eucalyptol effectively attenuated IL-1β secretion (Figure 6G). Furthermore, eucalyptol consistently suppressed nigericin-mediated IL-1β secretion (Figure 6H). However, MAPK activation in response to *P. acnes* was not changed (Figure 6I,J). Collectively, these results indicated that the effects of eucalyptol were consistent with those of LNE with regard to inhibiting *P. acnes*-induced inflammation signaling.

### 2.7. LNE Ameliorates P. acnes-Induced Skin Inflammation in vivo

**Figure 7:**
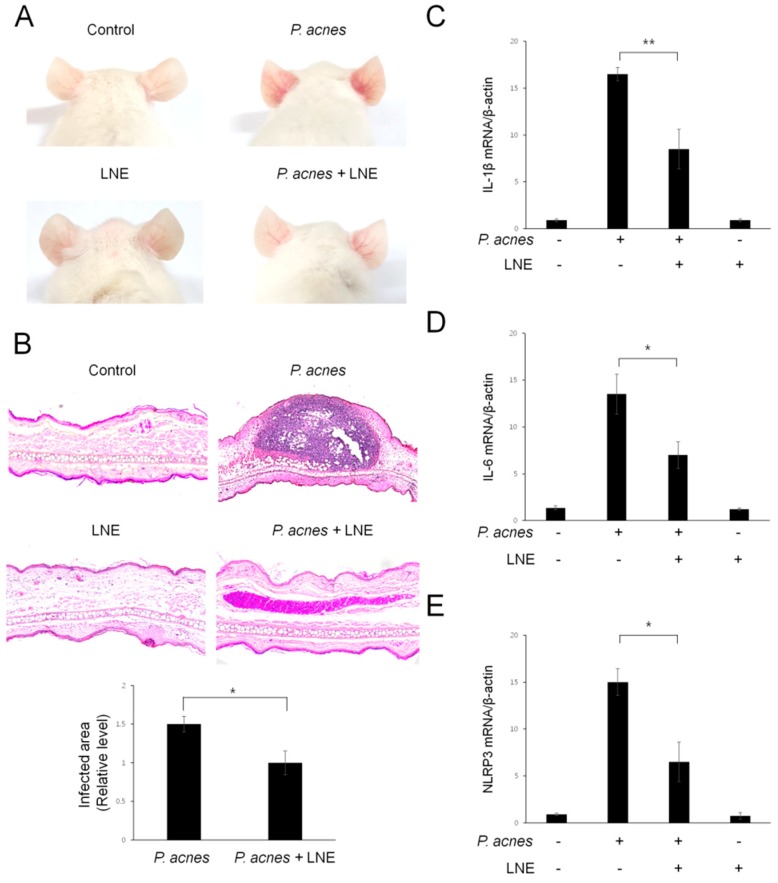
Inhibitory effects of LNE in a mouse acne model. Live *P. acnes* (1 × 10^8^ CFU) were inoculated into the ears of mice together with or without LNE (100 μg/mL). At 24 h after injection, the ears were photographed (**A**). (**B**) Formalin-fixed ear tissue sections were stained with H&E and the infected areas were quantified. Figures are representative of each study group (five mice per group). (**C**–**E**) The mRNA (IL-1β, IL-6, and NLRP3) levels were measured from ear tissues using quantitative real-time PCR. * *p* < 0.05, ** *p* < 0.01.

Next, we evaluated the pathophysiological effect of LNE in vivo using a mouse model. For this experiment, live *P. acnes* were intradermally injected into the ears of mice and histological changes were monitored. At 24 h after the *P. acnes* injection, the mouse exhibited cutaneous erythema, a typical symptom of ear inflammation (Figure 7A). However, *L. nobilis*-treated ears showed markedly reduced erythema, compared with ears injected with only *P. acnes* (Figure 7A). To further investigate the inflammatory responses in the skin, ear tissues were harvested and pathophysiological changes were monitored with hematoxylin and eosin (H&E) staining (Figure 7B). Inoculation of *P. acnes* induced swelling and an increase in the number of infiltrated inflammatory cells into the dermis (Figure 7B). In contrast, intradermal injection of LNE effectively attenuated *P. acnes*-mediated swelling and the granulomatous response (Figure 7B). To verify the reduction in ear inflammation by LNE treatment, we next quantified IL-1β, IL-6, and NLRP3 inflammasome mRNA levels, using quantitative real-time PCR (Figure 7C–E). The mRNA levels of these inflammatory molecules were consistently decreased by LNE treatment (Figure 7C–E). Collectively, these results suggest that LNE ameliorates *P. acnes*-induced skin inflammation in the mouse model.

## 3. Discussion

Acne is a common multifactorial disease with microbiological, hormonal, and immunological mechanisms. *P. acnes*, one of the normal skin flora, has long been suggested to be an etiological acne factor because it is increased in pilosebaceous unit of acne patients and an increase in the numbers of *P. acnes* elicits an innate immune response [2]. Although antibiotics have been used mainly for the reduction in numbers of *P. acnes*, the occurrence of the resistant strains of *P. acnes* increases the likelihood of therapeutic failure [22].

In response to various infection stimuli, macrophages express different types of pattern recognition receptors (PRRs), including surface receptors such as toll-like receptors (TLRs) and cytosolic pattern recognition receptors such as Nod-like receptors (NLRs). Several studies have shown that *P. acnes* interacts with TLR2 and TLR4 [23]. Activation of these pattern recognition receptors induces the secretion of proinflammatory cytokines and exacerbates skin inflammation in mice [24]. TLR2 have been found to be largely expressed in peribulbar and perifollicular macrophages in acne lesions, and TLR2-expressing cells have a correlation with the acne lesions [25]. Jugeau et al. also reported that the expression of TLR4 and TLR2 is increased in the epidermis of acne lesions [26]. NLRs are activated inside the cell and participate in inflammasome complexes, such as NLRP3 inflammasome [27]. The NLRP3 inflammasome is induced in response to the danger signal and regulates inflammation by triggering the secretion of proinflammatory cytokines, IL-1β [28]. Mutated NLRP3 causes autoinflammatory syndromes resulting in excess IL-1β production [29]. In addition, the NLRP3 inflammasome expression levels have been shown to be related with obesity and type II diabetes. Consistent with these reports, LNE and its constituent eucalyptol, attenuated *P. acnes*-induced IL-1β secretion, which correlated with the decrease of the NLRP inflammasome complex. However, further studies are required to assess whether NLRP3 is the only type of inflammasome in *P. acnes*-mediated IL-1β secretion.

Most TLRs lead to the activation of common downstream innate signaling cascades, such as MAPKs (JNK1/2, p38 MAPK, and ERK1/2) and NF-κB transcription [30]. MAPKs and NF-κB are activated by phosphorylation, which conveys the signal to the nucleus and then controls the expression of various inflammatory cytokines. In this study, LNE and eucalyptol suppressed *P. acnes*-induced NF-κB activation. However, MAPK activation was not altered by LNE. We, therefore, suggest that the anti-inflammatory effect of LNE occurs by inhibiting the NF-κB signaling cascade in BMMs.

The chemical composition of *L. nobilis* leaf has been reported in previous studies [20,31]. According to Cherrat et al., eucalyptol (1,8-cineole) and 2-carene are the main components [20]. In a study by Captuto et al., eucalyptol and sabinene were suggested as the main components [31]. Ozcan et al. also suggested eucalyptol as a main component of *L. nobilis* [21]. Many reports have suggested the anti-inflammatory properties of eucalyptol [32,33,34]. Eucalyptol exerts anti-inflammatory functions on acute pancreatitis via modulation of cytokines, oxidative stress, and NF-κB [32]. Eucalyptol protects from influenza virus-induced pneumonia in mice [33]. Eucalyptol also attenuates LPS-induced pulmonary inflammation in mice [34]. Eucalyptol mitigates amyloid-β (25–35)-induced inflammation in neuronally differentiated PC12 cells, which provide its potential in therapy in neurodegenerative disorder [35]. In addition to these reports, our study suggests a protective effect of LNE and its constituent eucalyptol in *P. acnes*-induced skin inflammatory signaling in BMMs.

To determine the physiological effect of LNE in *P. acnes*-induced inflammation, we used a well-known mouse model of acne, in which the pathological pattern is similar to that of human acne lesions [36]. We observed that injection of LNE into the mouse ear ameliorated *P. acnes*-induced inflammation, such as the granulomatous response, ear thickening, and corresponding inflammatory cytokine expression. We monitored the most common inflammation markers expressed in macrophages because tissue macrophages surrounding the pilosebaceous unit in acne lesions expressed high levels of TLR2 and correlated with the degree of inflammatory nature of the clinical lesions [25]. Our study is the first step to examine the possibility of natural product LNE for the suppression of *P. acnes*-mediated skin inflammation, using a mouse model. Based on our results, further studies could possibly check its application in clinical usages by comparing it with well-known antibiotics and standard materials.

**Figure 8:**
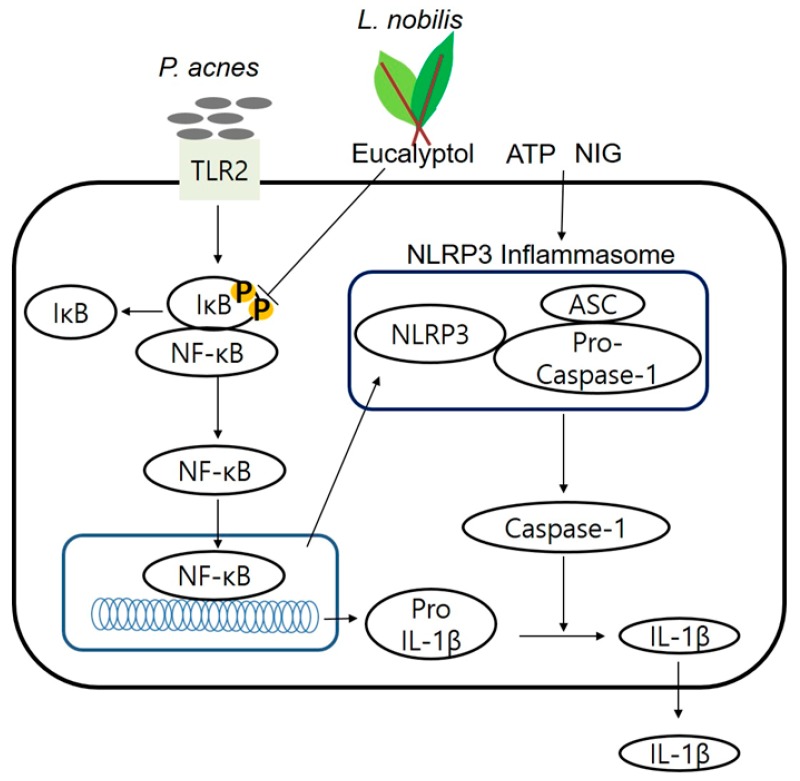
A model of *L. nobilis* leaf extract in the suppression of *P. acnes*-induced skin inflammation. *L. nobilis* exerts anti-inflammatory effects in acne lesions by inhibiting NF-κB signaling pathways.

In conclusion, our study was the first demonstration of the protective function of LNE on *P. acnes*-induced skin inflammation. LNE significantly reduced the expression of *P. acnes*-induced proinflammatory cytokine expression by suppressing NF-κB signaling pathways (Figure 8). In accordance with our results, the previous report has proved that eucalyptol penetrates from epidermis into stratum corneum in children and in adults [37]. We speculate that eucalyptol in LNE might penetrate into the skin and exert its anti-inflammatory effects. Our study suggests the potential of applying LNE and its major constituent eucalyptol to improve inflammatory skin disorder induced by *P. acnes*.

## 4. Materials and Methods

### 4.1. Materials

The anti-phospho-MAPKs (JNK, p38MAPK, and ERK) and anti-phospho-IκB antibodies were purchased from Cell Signaling Technology (Danvers, MA, USA). The anti-GAPDH antibody was purchased from Santa Cruz Biotechnology (Dallas, TX, USA). Nigericin was purchased from Tocris (Bristol, UK). Eucalyptol, ATP, and other chemicals were purchased from Sigma-Aldrich (St. Louis, MO, USA).

### 4.2. L. nobilis Extract (LNE)

LNE was prepared as described previously [38]. Briefly, the dried leaves of *L. nobilis* were purchased from the Hub Village (Ochang, Korea) and extracted with 70% ethanol for 3 h at room temperature. The filtered extract was then evaporated using a rotary evaporator (LABOTORA 4000eco, Heidolph Instruments GmbH&Co., Schwabach, Germany) to remove the solvent. The lyophilized sample was stored at –20 °C and was used after dissolving in dimethyl sulfoxide (DMSO) before each experiment. The presence of eucalyptol in the extract was identified by gas chromatography–mass spectrum (GC–MS) chromatogram (Agilent 7890A, Santa Clara, CA, USA).

### 4.3. P. acnes

*P. acnes* (KCTC3314) was obtained from the Korean Culture Center of Microorganisms (Seoul, Republic of Korea) and cultured in Reinforced Clostridial Medium (Merck Millipore, Darmstadt, Germany) under anaerobic conditions, using a Gas-Pak at 37 °C for 72 h. *P. acnes* were collected by centrifugation at 4500 rpm for 20 min at 4 °C, and the pellets were washed with PBS (Phosphate Buffered Saline).

### 4.4. Cell Culture

Bone marrow-derived macrophages (BMMs) were prepared as described previously [38]. Briefly, the progenitor cells were isolated from C57BL/6 mice and differentiated to BMMs in an L929 cell-conditioned medium (LCCM) [39,40]. The differentiated BMMs were cultured in RPMI1640 medium containing 10% heat-inactivated fetal bovine serum (FBS), 30% LCCM, penicillin, and streptomycin (Invitrogen, Carlsbad, CA), and were incubated with heat-killed *P. acnes*.

### 4.5. Western Blot Analysis

The cells lysates were separated on SDS-PAGE, and then transferred to nitrocellulose membrane. The membranes were blocked with 5% non-fat dried milk, and incubated with the indicated primary antibodies for 12 h. The bands were visualized with enhanced chemiluminescence system, and then quantified using ImageJ software 1.52a (NIH, Bethesda, MD).

### 4.6. Reporter Gene Analysis

NF-κB-Luc reporter and control Renilla-Luc reporter were transfected into HEK293 cells using a Lipofectamine (Invitrogen, Carlsbad, CA, USA). Luciferase activity was measured using a Dual-Luciferase Assays System (Promega, Madison, WI, USA).

### 4.7. Cell Counting

BMMs were plated in 12-well plates. The cells were treated with LNE for the indicated concentrations and then collected by centrifugation. The collected cells was mixed with a Trypan blue solution (Sigma-Aldrich, St. Louis, MO) and counted using a hemocytometer.

### 4.8. 3-(4,5-Dimethyl-2-Thiazolyl)-2H-Tetrazolium Bromide (MTT) Assay

The cells were incubated with MTT (Sigma-Aldrich, St. Louis, MO) at a final concentration of 1 mg/mL for 1 h at 37 °C and then lyzed in the solubilizing solution (50% dimethyl sulfoxide and 20% SDS, pH 4.8), overnight. Absorbance was measured at 570 nm and the viability of the cells was calculated as the percent relative to the control cells.

### 4.9. ELISA

The culture supernatants were collected and the concentrations of IL-1β were measured in accordance with the manufacturer’s instruction (R&D systems, Minneapolis, MN, USA).

### 4.10. Quantitative Real-Time PCR

Real-time PCR was performed as described previously [38]. cDNA was synthesized with M-MLV reverse transcriptase (Promega, Madison, WI) according to the manufacturer’s protocol, and then amplified with SYBR Green Master Mix (TOYOBO, Osaka, Japan), using the following primers: IL-1β, 5′-ACCTGTTCTTTGAGGCTGAC-3′ (forward) and 5′-CTTCTTTGGGTATTGTTTGG-3′ (reverse); IL-6, 5′-AGTTGCCTTCTTGGGACTGA-3′ (forward) and 5′-TTCTGCAAGTGCATCATCGT-3′ (reverse); NRLP3, 5′-ACCTGTTCTTTGAGGCTGAC-3′ (forward)and 5′-CTTCTTTGGGTATTGTTTGG-3′ (reverse) and β-actin, 5′-AGAGGGAAATCGTGCGTGAC-3′ (forward) and 5′-CGATAGTGATGACCTGACCGT-3′ (reverse). The values were analyzed using the CFX Manager ^TM^ (Bio-Rad, Hercules, CA, USA). All samples were run in triplicates and changes in target mRNA expression were normalized to β-actin.

### 4.11. In vivo Mouse Model

ICR mice were purchased from Doo Yeol Biotech (Seoul, Republic of Korea), and the mice were bred at the Animal Center of the Kangwon National University in a controlled environment. All procedures were performed according to protocols approved by the Institutional Animal Care and Use Committee (IACUC, KW-151002-1, Kangwon National University, Republic of Korea). Eight-week-old mice (five per group) were injected with live *P. acnes* (1 × 10^8^ CFU per 20 μL in PBS) intradermally into the ears with or without LNE (100 μg/mL). The mice were sacrificed at 24 h after the injection, and ear tissues were then collected for further analysis.

### 4.12. Histological Analysis

Isolated ear tissues were fixed in 4% formalin and embedded in paraffin. The sections (2–3 μm) were then stained with hematoxylin and eosin (H&E). Pathological changes were monitored by light microscopy (Olympus, Tokyo, Japan) and photographed.

### 4.13. Statistics

The statistical data were analyzed by SPSS (IBM, Armonk, NY, USA). The values are presented as the means ± standard deviations (SD) of three independent experiments. Comparisons between two groups were analyzed using Student’s *t*-test. Analysis of variance (ANOVA) followed by Bonferroni post hoc tests was performed for the multiple groups. A *p* < 0.05 was considered significant.
